# Isolation and Physicochemical Characterization of Laccase from* Ganoderma lucidum*-CDBT1 Isolated from Its Native Habitat in Nepal

**DOI:** 10.1155/2016/3238909

**Published:** 2016-10-16

**Authors:** Prabin Shrestha, Bishnu Joshi, Jarina Joshi, Rajani Malla, Lakshmaiah Sreerama

**Affiliations:** ^1^Central Department of Biotechnology, Tribhuvan University, Kirtipur, Nepal; ^2^Department of Chemistry and Earth Sciences, Qatar University, Doha, Qatar; ^3^Department of Chemistry and Biochemistry, St. Cloud State University, St. Cloud, MN 56301, USA

## Abstract

At present, few organisms are known to and capable of naturally producing laccases and white rot fungi are one such group. In the present study, three fungal species, namely,* Ganoderma lucidum*-CDBT1*, Ganoderma japonicum,* and* Lentinula edodes*, isolated from their native habitat in Nepal were screened for laccase production, and* G. lucidum*-CDBT1 was found to express highest levels of enzyme (day 10 culture media showed 0.92 IU/mg total protein or 92 IU/mL laccase activity with ABTS as substrate). Lignin extracted from rice straw was used in Olga medium for laccase production and isolation from* G. lucidum*-CDBT1. Presence of lignin (5 g/L) and copper sulfate (30 *μ*M) in the media increased the extracellular laccase content by 111% and 114%, respectively. The laccase enzyme produced by* G. lucidum*-CDBT1 was fractionated by ammonium sulfate and purified by DEAE Sepharose anion exchange chromatography. The purified enzyme was found to have a molecular mass of 43 kDa and exhibits optimal activity at pH 5.0 and 30°C. The isolated laccase was thermally stable for up to 70°C for 1 h and exhibited broad pH stability. The kinetic constants, *K*_*m*_, *V*_max_, and *K*_cat_, determined using 2,2′-azinobis-(-3-ethylbenzothiazoline-6-sulfonic acid) as substrate were found to be 110 *μ*M, 36 *μ*mol/min/mg, and 246 min^−1^, respectively. The isolated thermostable laccase will be used in future experiments for delignification process.

## 1. Introduction

Laccases (benzenediol: oxygen oxidoreductase, EC 1.10.3.2) are copper containing enzymes that catalyze one-electron oxidation of a wide variety of substrates such as diphenols, polyphenols, diamines, aromatic amines, and other electron rich compounds, using molecular O_2_ [1]. They belong to the family of proteins that include ascorbate oxidase, ceruloplasmin, and bilirubin oxidase. These enzymes are believed to utilize a free radical-catalyzed reaction mechanism in which the substrate forms an unstable free radical that further undergoes nonenzymatic reactions including hydration and disproportionation reactions [2].

Laccases are distributed among some plants [3, 4], fungi [3], and bacteria [5, 6]. Literature review shows that laccases are widely distributed among the prokaryotes. For example, laccases from* Azospirillum lipoferum *[7],* Marinomonas mediterranea *[8],* Streptomyces griseus *[9],* E. coli *[10],* Bacillus subtilis *[11], and many more bacteria have been purified and characterized. The crystal structures for several bacterial laccases have also been published [12–14]. Laccases are recognized for their wide spread applications including ethanol production, food industry, dye bleaching, paper and pulp processing, and production of value added chemicals from lignin [12]. Other applications of laccases include their use in detection of catecholamine neurotransmitters (dopamine, norepinephrine) and glucose dehydrogenase-coupled oxidation of morphine. Some fungal laccases have also been shown to detoxify fungal metabolites, for example, aflatoxin B1, and accordingly are useful in the field of food microbiology from the view point of food processing and preservation [15].

Among the fungi, the wood-rotting basidiomycetes produce different kinds of extracellular oxidoreductases including laccases, peroxidases, and oxidases that generate H_2_O_2_ [16]. The laccases from wood-rotting basidiomycetes are of significant interest as they are able to utilize a wide spectrum of carbon sources, including intermediates of lignin degradation, phenols, and heterocyclic compounds. Like bacterial laccases, the fungal enzymes are also involved in efficient degradation of lignin and various types of dyes such as azo, heterocyclic, reactive, and polymeric dyes [17–19]. Lignin is the most recalcitrant components in lignocellulosic biomass that encases the cellulose and hemicellulose, thus necessitating pretreatment of lignocellulosic biomass to liberate fermentable sugars to produce liquid biofuels. Given the lignin depolymerizing activity of laccases, they can serve as efficient biocatalysts for the pretreatment of lignocellulosic biomass to isolate fermentable sugars and other products [17–19].

Chemical pretreatment processes are available to release the fermentable sugars; however, they are expensive, difficult to operate, and environmentally unfriendly. Further, furan derivatives, weak acids, and phenolic compounds formed after lignin breakdown during chemical delignification have inhibitory effects on fermentation process [20, 21]. On the other hand, biological methods provide far safer and economic alternatives to obtain fermentable sugars from lignocellulosic biomass. One such alternative is depolymerization of lignin with the aid of laccases. There are numerous fungal species that grow on wooden logs and most of them secrete laccases to overcome the lignin barrier and thus obtain the energy from cellulose and hemicelluloses [3, 17–19]. The mountainous regions of Nepal are a rich source of fungi growing on logs [22]. In this regard, the Central Department of Biotechnology at Tribhuvan University, Kathmandu, Nepal, has collected, developed seed cultures and archived a number of fungal species. We believe that these fungal species have adapted to their natural environment of Nepal and some of them may be efficient laccase producers. Accordingly, as part of this study, we have screened three fungal species to identify potent laccase producers, optimized enzyme production conditions, and purified and characterized a laccase enzyme from* G. lucidum*-CDBT1.

## 2. Materials and Methods

### 2.1. Materials

 ABTS [2,2′-azino-bis(3-ethylbenzothiazoline-6-sulfonic acid)] and guaiacol were purchased from Hi-media Pvt. Ltd., New Delhi, India. DEAE Sepharose was purchased from Sigma Chemical Co., St. Louis, MO, USA. Protein molecular weight markers were purchased from Genei Pvt. Ltd., Bangalore, India. All other reagents and chemicals used were of analytical grade available locally.

### 2.2. Fungal Species Cultures

**Figure 1:**
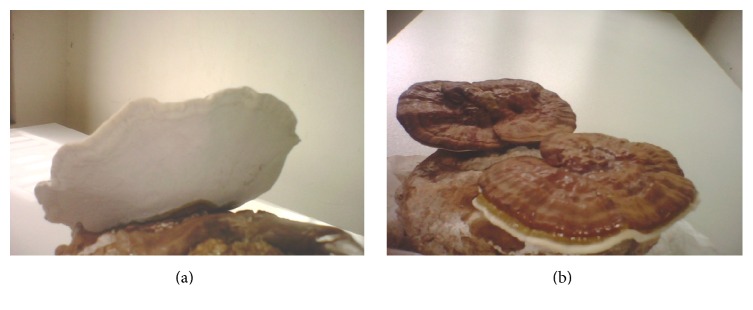
*Ganoderma lucidum*-CDBT1 fruiting bodies (courtesy of Central Department of Biotechnology): (a) ventral surface and (b) dorsal surface.

Pure cultures of* Ganoderma lucidum-*CDBT1 (Figure 1)*, Ganoderma japonicum-*CDBT2, and* Lentinula edodes-*CDBT3* (locally known as Shiitake) *preserved at 4°C were obtained from Central Department of Biotechnology, Tribhuvan University, Nepal. They were maintained by subculturing them in potato dextrose agar (PDA) Petri-plates by incubating the cultures at 25°C for 72 h in the case of* Ganoderma species* and 120 h for* Lentinula* species. Regarding the fungal species used herein, although not fully characterized, we do believe that they are novel strains adapted to growing in high altitudes of Nepal.

### 2.3. Screening of Fungal Species for Laccase Production

PDA-agar plates supplemented with guaiacol (0.02%) (wt/wt), 1-naphthol (5 mM), and tannic acid (0.5%) (wt/wt) and inoculated with various fungal cultures were used for screening of laccase production by the selected fungal species. All cultures were incubated at 25°C. Laccase secretion was monitored by visual color change in the plates, due to oxidation of screening agents, for several days [28, 29]. Biotic and abiotic cultures were used as positive and native controls, respectively. A reddish brown color was formed when secreted laccases react with guaiacol, a deep purple color was formed when they react with 1-naphthol, and a brown color was formed when laccases react with tannic acid. Guaiacol was added to the media before autoclaving, tannic acid was autoclaved separately before addition to the media, and 1-naphthol was autoclaved along with the media.

### 2.4. Lignin Isolation

Lignin was isolated from rice straw as described by Minu and associates [30]. Dried rice straw was powdered and oven dried overnight at 105°C. It was then hydrolyzed at 120°C for 60 min with 1% (wt/wt) sulfuric acid, and the resulting residue was then subjected to delignification process at 120°C for 60 min with alkaline peroxide [1.5% (wt/wt) NaOH and 0.5% (wt/wt) H_2_O_2_]. In all hydrolysis steps, the total solids used were 10% (wt/wt) and remaining 90% included 89% (wt/wt) water and 1% (wt/wt) sulfuric acid. In the delignification step, the total solids used were 10% (wt/wt) acid treated rice straw and remaining 90% was made up of 88% (wt/wt) water and 1.5% (wt/wt) NaOH and 0.5% (wt/wt) H_2_O_2_. The solubilized lignin also known as black liquor was separated from the solids. Lignin was isolated from the black liquor by two-step treatments. In the first step, the pH was decreased to 7.0 and vacuum filtered to remove silica. In the second step, the liquid was further titrated with acid to drop the pH to 3.0 and incubated overnight to precipitate lignin. The precipitated lignin was washed, vacuum filtered, and dried.

### 2.5. Laccase Enzyme Assay

Laccase enzyme activity was measured at room temperature using ABTS as substrate (1 mM solution prepared in 0.1 M of sodium acetate buffer, pH 5.0). A typical reaction mixture consisted of 350 *μ*L of enzyme preparation (isolated or purified and appropriately diluted) and 350 *μ*L of 1 mM ABTS and the final volume was adjusted to 1.15 mL with 0.1 M of sodium acetate buffer, pH 5.0. Reaction was started by the addition of enzyme and oxidation of ABTS was monitored spectrophotometrically at 420 nm for 90 seconds using quartz cuvettes. The amount of enzyme required to oxidize 1 *μ*mol ABTS per minute is considered equivalent to 1 unit of enzyme activity (*ε*420 = 36000 M^−1 ^cm^−1^ and path length l = 1 cm) [31].

### 2.6. Optimization of Laccase Production from* Ganoderma lucidum*-CDBT1

Modified Olga medium [4 g/L glucose, 3 g/L peptone, 0.6 g/L K_2_HPO_4_, 0.4 g/L KH_2_PO_4_, 1 mg/L ZnSO_4_, 5 mg/L FeSO_4_, and 0.5 g/L MnSO_4_, 0.5 mg/L MgSO_4_, 0.5 mg/L CuSO_4_, and 5 g/L lignin] was used for optimization and production of laccase from* G. lucidum*-CDBT1 [32]. Four parameters, namely, (i) presence of lignin (control experiments contained no lignin in the media), (ii) temperature (range 20 to 50°C varied at an interval of 10°C), (iii) pH (varied between pH 3.0 and 8.0 at an interval of 0.5 pH units), and (iv) copper sulfate concentration (varied between 10 *μ*M to 50 *μ*M at an interval of 10 *μ*M; control experiments contained no copper sulfate in the media), were used to optimize laccase production. The pH of the media was varied using 0.1 M sodium acetate (pH 3–6), 0.1 M sodium phosphate (pH 6.5–7.5), or 0.1 M Tris-HCl (>pH 7.5). Each experimental culture consisted of 50 mL modified Olga media and 5 discs of actively growing* G. lucidum*-CDBT1 mycelium (5-day-old culture, 7 mm diameter discs) under varied conditions (above) and they were incubated in an orbital shaker incubator at 160–200 rpm at room temperature (~25°C) except when the temperatures were varied. Preliminary experiments suggested a concentration of 5 g/L lignin was optimal for the production of laccase; accordingly, all cultures, with the exception of the controls, were grown in the presence of 5 g/L of lignin isolated from rice straw. Laccase enzyme activity in the culture media was monitored at 48-hour (2-day) intervals.

### 2.7. Purification of Laccase

Laccase secreted into the culture media was purified in three different steps. First, the cultures were vacuum filtered using Whatman number 1 filter paper. Thus obtained supernatant was centrifuged at 10,000 ×g for 10 min to remove any debris and the clear supernatant was further used for purification process [33]. Second, the supernatant was subjected to ammonium sulfate fractionation (40% to 80%). The precipitated protein was isolated by centrifugation (10,000 ×g/10 min). The precipitates were reconstituted in 0.1 M sodium acetate buffer, pH 5.0, and screened for the presence of laccase activity as described above. The fractions with high laccase activity were subjected to dialysis overnight against 0.1 M sodium phosphate buffer pH 7.0. Finally, the desalted enzyme factions were subjected to a DEAE Sepharose anion exchange column chromatography at pH 7.0 as described previously [25]. The protein fractions collected were analyzed for laccase activity, and the fractions with high laccase activity were pooled and subjected to SDS- and native-PAGE to assess its purity and determine its molecular mass.

### 2.8. Electrophoresis

Molecular mass of laccase was determined by SDS-PAGE using standard molecular mass markers (Genei India Pvt. Ltd.) according to Laemmli's method [34]. Polyacrylamide gels (6% stacking gel and 10% separating gel) loaded with denatured and reduced protein preparations were subjected to electrophoresis at 25 mA for 3 hours at room temperature. Proteins were visualized on PAGE gels using EZ-Visi Blue protein staining solution [5]. Native-PAGE was also as described above except the gels were run at 4°C; protein samples were not denatured or reduced, and SDS was omitted from the gels. The native-PAGE gels were stained with 5 mM ABTS solution and 1% guaiacol solutions for 15 min to visualize laccase bands on the gels.

### 2.9. Protein Quantitation

Protein content in various preparations used in this study were determined by Bradford method [35] using BSA as standard.

### 2.10. Physicochemical Characterization of Laccase

The pH and temperature optima for the purified laccase were determined as follows. Purified laccase enzyme activity was determined by varying the pH 2.0–8.0 or the temperature (20–80°C) of the reaction under standard assay conditions described above. To investigate the pH stability, the purified enzyme was preincubated in buffers of pH 2.0–8.0 at room temperature for up to 240 min before their enzyme activities were determined. Similarly, to investigate the thermal stability, the purified enzyme (dialyzed against 0.1 M sodium acetate buffer pH 5.0) was incubated in water baths at various temperatures between 30 and 80°C for up to 180 min. An aliquot of the enzyme was withdrawn every 30 min and assayed for enzyme activity as above. Michaelis–Menten kinetics were used to determine *K*_*m*_ and *V*_max_ using ABTS as the substrate (0.01 to 10 mM) and the kinetic constants (*K*_*m*_ and *V*_max_) were derived from Lineweaver-Burk plots [28].

### 2.11. Data Analysis

Microsoft Excel and Prism GraphPad V 5.00 computer programs were used for data analysis. All values reported are average of triplicate experiments.

## 3. Results

### 3.1. Screening Fungal Species for Laccase Activities

**Figure 2:**
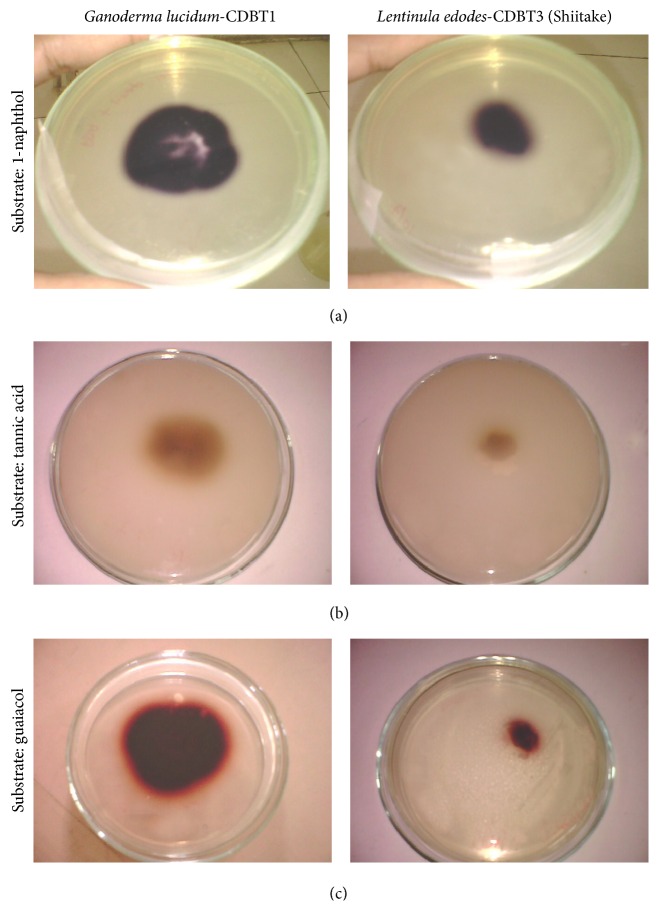
Screening of* Ganoderma lucidum-*CDBT1 and* Lentinula edodes-*CDBT3* (Shiitake) *for secretion of laccase. The experimental conditions were as described in Materials and Methods and stained with 1-naphthol (a), tannic acid (b), and guaiacol (c).

Three white rot fungal species, namely,* G. lucidum*-CDBT1,* G. japonicum*-CDBT2, and* L. edodes*-CDBT3 (*Shiitake)*, were screened for the secretion of laccase using PDA plates supplemented with 1-naphthol, tannic acid, and guaiacol [28, 29]. The oxidative polymerization of guaiacol forming reddish brown zones in the medium, oxidation of tannic acid to brown color [29], and oxidation of 1-naphthol to a deep purple complex [28] are a visual confirmation for the presence/secretion of laccase enzyme. In this test, all species tested, that is,* G. lucidum*-CDBT1,* G. japonicum*-CDBT2, and* L. edodes*-CDBT3 (*Shiitake),* gave visual colors described above indicating secretion of extracellular laccase into the PDA medium* G. lucidum-*CDBT1 formed the largest zone of coloration (Figure 2), followed by* L. edodes-*CDBT3* (Shiitake) *(Figure 2)* and G. japanicum-*CDBT2* (data not presented).* Accordingly,* G. lucidum-*CDBT1 was chosen for the further study.

### 3.2. Optimization of Laccase Production Media: Effect of Lignin, Copper Sulfate, Temperature, and pH

**Figure 3:**
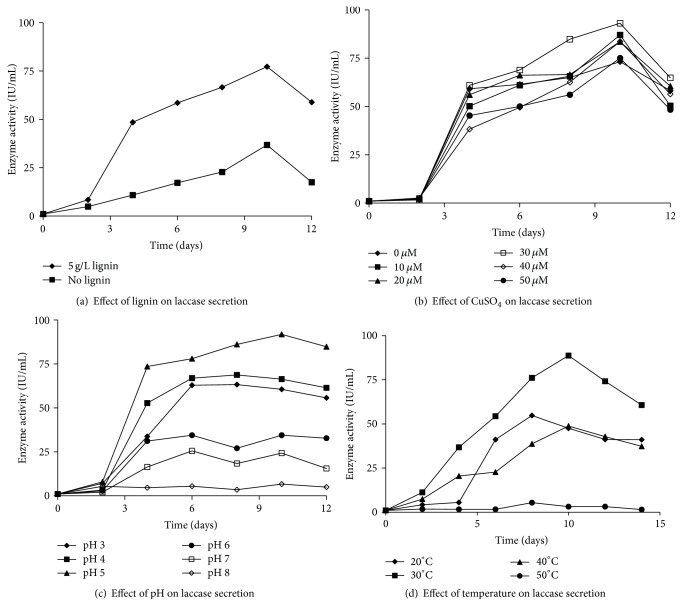
Optimization of experimental conditions for laccase production in* Ganoderma lucidum*-CDBT1. Modified Olga medium was used for* G. lucidum*-CDBT1 under various conditions shown in (a)–(d) as described in Materials and Methods. Experiments described in (a), (b), and (c) were performed at room temperature (~25°C). The modified Olga medium used to grow* G. lucidum* (experiments described in (a), (c), and (d)) contained 0.5 g/L CuSO_4_ and 5 g/L lignin (experiments described in (b), (c), and (d)). (a) Effect of 5 g/L lignin on laccases secretion, (b) effect of CuSO_4_ on laccase secretion, (c) effect of pH of the media on secretion of laccase, and (d) effect of incubation temperature on the secretion of laccases. Enzyme activity was determined as described in Materials and Methods. With the exception of the experiments involving the effect of media pH on secretion of laccase, all other experiments were performed at pH 5.0.

Presence of lignin and copper sulfate is known to promote production/secretion of laccase. In this regard the culture media were supplemented with 5 g/L lignin (isolated from rice straw). This resulted in 111% increase in the secretion of laccase as compared to a control culture supplemented with glucose (Figure 3). The effect of copper sulfate on laccase production was determined by varying its concentration (10–50 *μ*M) in the culture medium. Maximum laccase production (93 IU/mL on 10th day; a 114% increase as compared to the blank) was obtained at a concentration of 30 *μ*M copper sulfate (Figure 3).* G. lucidum*-CDBT1 cultures were further subjected to temperature and pH optimization studies to determine the conditions required for the optimal secretion of laccase. The optimal temperature and pH needed to produce high levels of laccase were found to be 30°C (89 IU/mL on day 10) and pH 5.0 (92 IU/mL on day 10), respectively (Figure 3). Secretion of detectable levels of the enzyme activity begins as early as day 2 and reaches a maximum at around day 10. Enzyme activity decreased sharply as the pH increased from 5.0 towards the neutral range or the temperature increased towards 50°C (Figure 3).

### 3.3. Purification of Laccase

**Figure 4:**
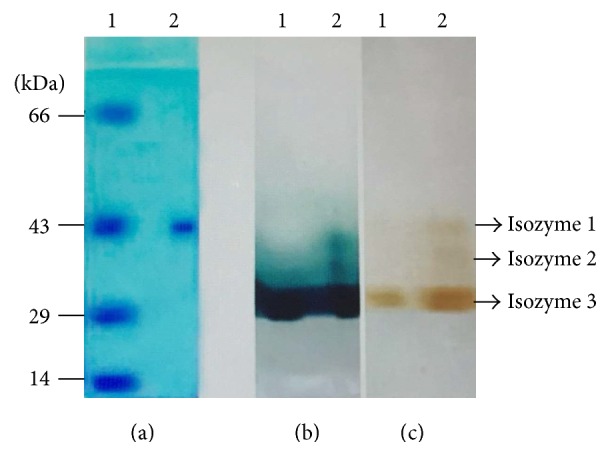
PAGE analysis of laccase isolated from* Ganoderma lucidum*-CDBT1. (a) SDS-PAGE analysis of purified laccase (Lane 1: marker proteins; Lane 2: purified laccase protein); (b) and (c) native-PAGE analysis of laccase purified (Lane 1) or isolated from culture medium (Lane 2). (b) The gel was stained with guaiacol as substrate and (c) the gel was stained with ABTS as substrate.

The culture filtrate obtained from culture media subjected to ammonium sulfate precipitation led to enrichment of laccase in the 70% ammonium sulfate faction. The precipitate dissolved in sodium acetate buffer, pH 5.0, and dialyzed overnight was further purified by DEAE Sepharose anion exchange column chromatography, Table 1. The column was developed using a linear gradient of 0 to 0.1 M sodium chloride at pH 7.0. The peak fractions were pooled and analyzed by native- and denaturing-PAGE (Figure 4). The ion-exchange chromatography purified laccase protein pool showed the presence of a single protein band on SDS-PAGE gels, with a molecular weight of 43 kDa (Figure 4(a)). Native-PAGE gels stained with ABTS and guaiacol as substrates also indicated the presence of one isozyme of laccase (Figures 4(b) and 4(c)) in the purified faction. The extracts, as judged by native-PAGE, showed the presence of 3 laccase isozymes and the purified enzyme corresponds to isozyme 3 (the major isoform produced).

### 3.4. Partial Characterization of Purified Laccase: pH and Temperature Optima and pH and Thermal Stability

**Figure 5:**
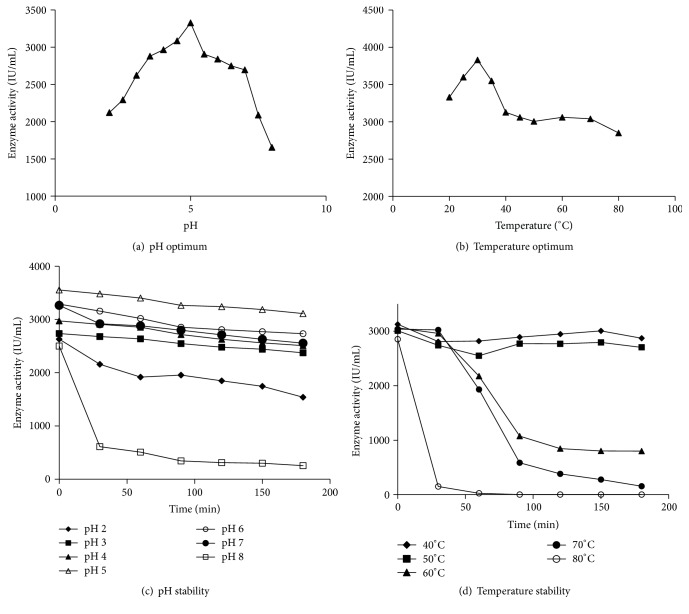
pH and temperature optimum and pH and thermal stability of purified laccase. The experimental conditions were as described in Materials and Methods. Experiments described in (a) and (c) were performed at room temperature (~25°C). The experimental results depicted in (a) and (c) (pH optimum and pH stability) were performed using buffers of various pH specified, whereas the temperature optimum and thermal stability was performed at pH 5 (0.1 M sodium acetate, pH 5.0).

The influence of pH and temperature on laccase activity was determined by varying pH of the reaction mixture from pH 2.0 to 8.0, while the influence of temperature on laccase activity was determined by varying the temperature of the reaction between 20 and 80°C and at pH 5.0 (pH optima). The pH and temperature optima for laccases were found to be pH 5.0 and 30°C, respectively (Figures 5(a) and 5(b)). Laccase was active in a wide range of pH values. At pH values above pH 5.0, the enzyme activity decreased gradually by 50% at pH 8 (Figure 5(a)). Laccase activity was relatively stable in the range of pH 3.0–7.0. The enzyme activity declined when the temperature was increased from 30 to 80°C (Figure 5(b)). Purified laccase was incubated in buffers of varying pH between pH 2.0 and 8.0 for up to 180 min to determine its pH stability. Similarly, the purified enzyme dissolved in 0.1 M sodium acetate, pH 5.0, was incubated at varying temperatures (40–80°C) for up to 180 min to determine its thermal stability. Laccase was found to be most stable at pH 5.0 and 30°C (Figures 5(c) and 5(d)). Laccase activity decreased significantly (93%) after 180 min at pH 8.0 and it was fairly stable between pH 3.0 and 7.0 for up to 180 min. While the enzyme was relatively stable below 50°C, activity decreased significantly when the temperature was 70°C or higher. In fact, laccase activity was completely lost within an hour at 80°C (Figures 5(c) and 5(d)).

### 3.5. Determination of Kinetic Parameters

The kinetic parameters (*K*_*m*_, *V*_max_, and *K*_cat_) for the purified laccase enzyme were determined using varied concentrations of ABTS as substrate and they were found to be 110 *μ*M, 36 *μ*mol/min/mg, and 246 min^−1^, respectively.

## 4. Discussion

Some of the most important factors that influence secretion of laccase into culture media by* Ganoderma* species include (i) pH and temperature for their growth, (ii) addition of suitable amounts of sugars, for example, glucose (carbon source), (iii) addition of lignin (inducer), and (iv) an appropriate concentration of copper sulfate (inducer). The optimal pH conditions under which fungal laccases are secreted into culture media vary between pH 3.0 and 7.0. This also depends on the type of substrate used in the activity assays. For example, when ABTS is used as substrate, the pH optima are more in acidic range, pH 3.0–5.0 [1]. In our study, laccase secretion was found to be maximal at pH 5.0. Accordingly, all of the cultures used for the production of laccase from* G. lucidum*-CDBT1 in this study were performed at pH 5.0. Further, the enzyme was secreted into the culture media over a wide range of pH, suggesting versatility of the enzyme for applications in many biotechnological processes. The decrease in activity at higher pH is most probably due to the binding of a hydroxide anion to type 2 and 3 copper centers in laccase, which in turn inhibits the binding of oxygen to the copper centers and thus decreased laccase activity [35, 36].

*G. lucidum*-CDBT1 cultures used in this study secreted maximal laccase into the culture medium at 30°C. This has been supported by the fact that* G. lucidum* is a mesophilic fungus and others have shown that* G. lucidum* secretes maximal laccase at 25°C [25].

Addition of glucose to culture media has been shown to influence laccase synthesis [37]. The concentration of glucose used in these studies varied from 0.1 to 1% [38]. The* G. lucidum*-CDBT1 cultures used herein were optimized to grow in relatively low concentrations (0.4%) of glucose containing media, and under these conditions* G. lucidum*-CDBT1 culture produced relatively high levels of laccase, while high concentrations of sugars may satisfy the nutrient demands of* G. lucidum *for growth but do not necessarily promote optimal secretion of laccase. In certain species, for example,* Trametes pubescens, *laccase production was, in fact, inhibited by high concentrations of glucose [39]. Additionally, it has also been shown that high concentrations of glucose trigger synthesis of extracellular polysaccharides that will interfere with the extraction of laccase from culture media [40]. Given the above factors, 0.4% glucose was used while optimizing our cultures.

Addition of lignin to culture media has also been shown to enhance laccase secretion [41]. Our cultures were optimized to grow and secrete maximal amount of laccase into culture media at concentrations of 5 g/L lignin (0.5%). Such concentrations resulted in two-fold increase in laccase activity in cultures, as compared to the culture media containing only 0.4% glucose. The latter may be due to the synthesis of inducible laccase isozymes, which in turn is believed to be because of increased secondary metabolism. Sharma and associates [41] have also observed an increase in laccase yield in a* Ganoderma* sp., rckk-02, when cultured in the presence of increasing concentrations of lignin. Other compounds shown to increase laccase production in cultures include veratryl alcohol, syringic acid, and 2,5-xylidine [41–43]. We used peptone as a nitrogen source which is known to enhance laccase synthesis [38]. Due to the complex composition of peptones, they provide a wide range of benefits to the cells and cell performance. Copper ions (Cu^2+^) are part of the active site of laccases; accordingly, CuSO_4_ is most frequently supplemented with growth media to enhance laccase production in fungi. Cu^2+^ ions are thus crucial for the synthesis of catalytically active laccase protein. Further, Cu^2+^ ions also induce the expression of certain genes including laccase gene [44]. The promoter region of the genes encoding for laccase contains various recognition sites specific for xenobiotics and heavy metals [3, 45]. It has been demonstrated that the* Pleurotus* laccase genes* poxc* and* poxa1b* are transcriptionally induced by Cu^2+^ ions [45, 46]. Relatively high concentrations of Cu^2+^ have also been shown to suppress laccase production which could be due to activation of defense mechanisms [42]. In our experiments, the optimal concentration of Cu^2+^ required to express highest levels of laccases was 30 *μ*M. This is consistent with other studies [45, 46]. In addition to Cu^2+^, distinct organic inducers with structural similarities or relationships with lignin are often used to induce the expression of laccase [47].

Many fungal laccase isozymes have been purified and their molecular properties have been summarized [3]. The laccase enzyme purified from* G. lucidum*-CDBT1 had a molecular mass of 43 kDa and exhibited *K*_*m*_, *V*_max_, and *K*_cat_ values of 0.110 mM, 36 *μ*mol/min/mg, and 246 min^−1^, respectively. The *K*_*m*_ values for laccases reported previously ranged from 0.1 to 3.7 mM with ABTS as substrate [23]. The *K*_*m*_ and *V*_max_ values of recombinant laccase heterologously expressed in* Pichia pastoris* using ABTS substrate were found to be 0.521 mM and 19.65 *μ*mol/min/mg , respectively [3, 23, 48]. *K*_*m*_ reported in this work is lower than most other reported values suggesting this enzyme has higher affinity towards nonphenolic substrate ABTS [47]. This is as expected as these enzymes originate from different organisms and their physicochemical characteristics have been shown to be different. The other physicochemical characteristics, for example, pH and temperature optima, of the laccase isozyme isolated from* G. lucidum-*CDBT1 were similar but not necessarily identical to those previously reported [25, 47]. The isolated enzyme was thermally stable (70°C for up to 1 h) and exhibited broad pH stability (pH 3.0–7.0).

Fungal species express a number of different laccase isozymes with molecular masses ranging from 24 to 80 kDa [40], and this variation could be attributed to the different ecological origins of the species or different culture conditions under which various isozymes were expressed and/or secreted. In particular, presence of the inducers in the media seems to play an important role in which laccase isozymes were secreted into the medium. For example,* P. pulmonarius *produced three laccase isozymes, two of which (lcc1 and lcc2) were constitutive and the 3rd is inducible (lcc3); lcc3 was detected only when the fungus was cultured in the presence of inducers [48]. This also appears to be true among various strains of* G. lucidum,* Table 2. In this study, native-PAGE analysis suggested the presence of three laccase isozymes in* G. lucidum*-CDBT1. As judged by native-PAGE two of the isozymes were minor components whereas the 3rd was a major inducible isozyme. The major isozyme has been successfully purified in this study. This enzyme has characteristics similar to the enzyme isolated by Ko and associates [25] although the molecular mass is different. Molecular weight of the laccase isozyme-3 (Figure 4), reported herein (43 kDa as judged by SDS-PAGE), is in agreement with the value reported by Murugesan and associates [49] based on SDS- and native-PAGE of crude extracts stained for enzyme activity. This study [49] did not undertake physicochemical characterization of the enzyme. The minor isoforms that we have observed in native-PAGE gels are being isolated using enrichment processes in an ongoing study. Ko and associates [25] have, in fact, reported the presence of three laccase isozymes in* G. lucidum *whose molecular masses ranged between 40 and 68 kDa [42]. The number of laccase isozymes secreted by other fungal species also varies. For example,* T. multicolor *is reported to secrete five isozymes [50] whereas* P. ostreatus* secretes eight different laccase isozymes in response to Cu^2+^ ions [51]. The number of laccase isozymes expressed/produced by different strains of the same species was also different [23–27, 37]. In this regard, we have summarized some physicochemical characteristics of several laccases isolated from a number of different strains of* G. lucidum*, Table 2. Accordingly, it appears that the laccase isozyme 3 is isolated and purified in this study, although having similar enzyme characteristics, its molecular mass is different. Further molecular characterization, for example, sequence determination and its comparison to other know sequences, is required to call this a novel enzyme.

In conclusion, we have successfully cultured and optimized culture conditions, induced expression, and purified and characterized a laccase in* G. lucidum-*CDBT1 isolated from its native habitat in Nepal. The isozyme characterized from* G. lucidum-*CDBT1 appears to be different from those reported among various strains of* G. lucidum*. Identification of minor isoforms of laccases in* G. lucidum-*CDBT1 and various laccases present in other organisms screened in this study, that is,* G. japonicum* and* L. edodes,* and the use of these enzymes to depolymerize lignin to expose cellulose and hemicellulose fibers from lignocellulosic biomass for efficient hydrolysis and fermentation of sugars are being studied in our laboratory.

## Figures and Tables

**Table 1:** Summary of purification of laccase from *Ganoderma lucidum*-CDBT1^*∗*^.

Enzyme	Total activity (U)	Protein (*μ*g/mL)	Specific activity (U/mg)	Fold-purification
Culture media-filtrate	97.22	105.7	0.92	1.0
Ammonium sulfate faction (after overnight dialysis)	3115.74	35.9	86.85	94.4
DEAE-anion exchange chromatography	2662.04	13.3	201.00	218.0

^*∗*^Preparation of the culture media-filtrate containing laccase and its ammonium sulfate fractionation and DEAE-anion exchange chromatography to isolate laccase were as described in Materials and Methods. Laccase enzyme was monitored using 1 mM ABTS as substrate at room temperature (~25°C) and protein concentrations in various factions were determined by Bradford assay described in Materials and Methods.

**Table 2:** A comparison of the physicochemical characteristics of laccases isolated from several strains of *Ganoderma lucidum*.

*G. lucidum* strain	Mol. mass (kDa)	pH optimum	*K*_*m*_ with ABTS as substrate (mM)	Country of origin	Reference
Unknown	68	3.0	0.114	China	[23]
CBS229.93	62.5	5.0	0.107	Netherlands	[24]
Galc3	65–68	3.5	0.037	South Korea	[25]
IBL-05	38.3	5.0	0.047	India	[26]
MDU-7	24–66	5.0	0.026–0.029	India	[27]
CDBT1	43	5.0	0.110	Nepal	This work

## References

[1] Madhavi V., Lele S. S. (2009). Laccase: properties and applications. *BioResources*.

[2] Claus H. (2004). Laccases: structure, reactions, distribution. *Micron*.

[3] Baldrian P. (2006). Fungal laccases-occurrence and properties. *FEMS Microbiology Reviews*.

[4] Shekhar R. S., Sehgal S., Kamthania M., Kumar A. (2011). Laccase: microbial sources, production, purification, and potential biotechnological applications. *Enzyme Research*.

[5] Hildén K., Hakala T. K., Maijala P., Lundell T. K., Hatakka A. (2007). Novel thermotolerant laccases produced by the white-rot fungus *Physisporinus rivulosus*. *Applied Microbiology and Biotechnology*.

[6] Sharma P., Goel R., Capalash N. (2007). Bacterial laccases. *World Journal of Microbiology and Biotechnology*.

[7] Diamantidis G., Effosse A., Potier P., Bally R. (2000). Purification and characterization of the first bacterial laccase in the rhizospheric bacterium *Azospirillum lipoferum*. *Soil Biology and Biochemistry*.

[8] Solano F., García E., Perez D., Sanchez-Amat A. (1997). Isolation and characterization of strain MMB-1 (CECT 4803), a novel melanogenic marine bacterium. *Applied and Environmental Microbiology*.

[9] Endo K., Hayashi Y., Hibi T., Hosono K., Beppu T., Ueda K. (2003). Enzymological characterization of EpoA, a laccase-like phenol oxidase produced by Streptomyces griseus. *Journal of Biochemistry*.

[10] Kim C., Lorenz W. W., Hoopes J. T., Dean J. F. D. (2001). Oxidation of phenolate siderophores by the multicopper oxidase encoded by the *Escherichia coli* yacK gene. *Journal of Bacteriology*.

[11] Muthukumarasamy N. P., Jackson B., Joseph Raj A., Sevanan M. (2015). Production of extracellular laccase from *Bacillus subtilis* MTCC 2414 using agroresidues as a potential substrate. *Biochemistry Research International*.

[12] Giardina P., Faraco V., Pezzella C., Piscitelli A., Vanhulle S., Sannia G. (2010). Laccases: a never-ending story. *Cellular and Molecular Life Sciences*.

[13] Gunne M., Höppner A., Hagedoorn P.-L., Urlacher V. B. (2014). Structural and redox properties of the small laccase Ssl1 from *Streptomyces sviceus*. *The FEBS Journal*.

[14] Majumdar S., Lukk T., Solbiati J. O. (2014). Roles of small laccases from *Streptomyces* in lignin degradation. *Biochemistry*.

[15] Alberts J. F., Gelderblom W. C. A., Botha A., van Zyl W. H. (2009). Degradation of aflatoxin B1 by fungal laccase enzymes. *International Journal of Food Microbiology*.

[16] Wesenberg D., Kyriakides I., Agathos S. N. (2003). White-rot fungi and their enzymes for the treatment of industrial dye effluents. *Biotechnology Advances*.

[17] Murugesan K., Yang I.-H., Kim Y.-M., Jeon J.-R., Chang Y.-S. (2009). Enhanced transformation of malachite green by laccase of *Ganoderma lucidum* in the presence of natural phenolic compounds. *Applied Microbiology and Biotechnology*.

[18] Kirk T. K., Farrell R. L. (1987). Enzymatic ‘combustion’: the microbial degradation of lignin. *Annual Review of Microbiology*.

[19] Gianfreda L., Xu F., Bollag J.-M. (1999). Laccases: a useful group of oxidoreductive enzymes. *Bioremediation Journal*.

[20] Joshi B., Bhatt M. R., Sharma D., Joshi J., Malla R., Sreerama L. (2011). Lignocellulosic ethanol production: current practices and recent developments. *Biotechnology and Molecular Biology Reviews*.

[21] Almeida J. R. M., Modig T., Petersson A., Hähn-Hägerdal B., Lidén G., Gorwa-Grauslund M.-F. (2007). Increased tolerance and conversion of inhibitors in lignocellulosic hydrolysates by *Saccharomyces cerevisiae*. *Journal of Chemical Technology and Biotechnology*.

[22] Christensen M., Bhattarai S., Devkota S., Larsen H. O. (2008). Collection and use of wild edible fungi in Nepal. *Economic Botany*.

[23] Ding Z., Peng L., Chen Y. (2012). Production and characterization of thermostable laccase from the mushroom, *Ganoderma lucidum*, using submerged fermentation. *African Journal of Microbiology Research*.

[24] Manavalan T., Manavalan A., Thangavelu K. P., Heese K. (2013). Characterization of optimized production, purification and application of laccase from Ganoderma lucidum. *Biochemical Engineering Journal*.

[25] Ko E.-M., Leem Y.-E., Choi H. T. (2001). Purification and characterization of laccase isozymes from the white-rot basidiomycete *Ganoderma lucidum*. *Applied Microbiology and Biotechnology*.

[26] Bilal M., Asgher M. (2016). Enhanced catalytic potentiality of *Ganoderma lucidum* IBL-05 manganese peroxidase immobilized on sol-gel matrix. *Journal of Molecular Catalysis B: Enzymatic*.

[27] Kumar A., Sharma K. K., Kumar P., Ramchiary N. (2015). Laccase isozymes from Ganoderma lucidum MDU-7: isolation, characterization, catalytic properties and differential role during oxidative stress. *Journal of Molecular Catalysis B: Enzymatic*.

[28] More S. S., Renuka P. S., Pruthvi K., Swetha M., Malini S., Veena S. M. (2011). Isolation, purification, and characterization of fungal laccase from *Pleurotus* sp.. *Enzyme Research*.

[29] Kiiskinen L.-L., Rättö M., Kruus K. (2004). Screening for novel laccase-producing microbes. *Journal of Applied Microbiology*.

[30] Minu K., Jiby K. K., Kishore V. V. N. (2012). Isolation and purification of lignin and silica from the black liquor generated during the production of bioethanol from rice straw. *Biomass and Bioenergy*.

[31] Wolfenden B. S., Willson R. L. (1982). Radical-cations as reference chromogens in kinetic studies of one-electron transfer reactions. *Journal Chemical Society Perkin Transactions*.

[32] Koroljova-Skorobogat'ko O. V., Stepanova E. V., Gavrilova V. P. (1998). Purification and characterization of the constitutive form of laccase from the basidiomycete *Coriolus hirsutus* and effect of inducers on laccase synthesis. *Biotechnology and Applied Biochemistry*.

[33] Han M.-J., Choi H.-T., Song H.-G. (2005). Purification and characterization of laccase from the white rot fungus *Trametes versicolor*. *Journal of Microbiology*.

[34] Laemmli U. K. (1970). Cleavage of structural proteins during the assembly of the head of bacteriophage T4. *Nature*.

[35] Bradford M. M. (1976). A rapid and sensitive method for the quantitation of microgram quantities of protein utilizing the principle of protein-dye binding. *Analytical Biochemistry*.

[36] Xu F. (1997). Effects of redox potential and hydroxide inhibition on the pH activity profile of fungal laccases. *Journal of Biological Chemistry*.

[37] Teerapatsakul C., Abe N., Bucke C., Kongkathip N., Jareonkitmongkol S., Chitradon L. (2007). Novel laccases of *Ganoderma* sp. KU-Alk4, regulated by different glucose concentration in alkaline media. *World Journal of Microbiology and Biotechnology*.

[38] Kumar V. V., Kirupha S. D., Periyaraman P., Sivanesan S. (2011). Screening and induction of laccase activity in fungal species and its application in dye decolorization. *African Journal of Microbiology Research*.

[39] Galhaup C., Wagner H., Hinterstoisser B., Haltrich D. (2002). Increased production of laccase by the wood-degrading basidiomycete *Trametes pubescens*. *Enzyme and Microbial Technology*.

[40] Eggert C., Temp U., Eriksson K.-E. L. (1996). The ligninolytic system of the white rot fungus *Pycnoporus cinnabarinus*: purification and characterization of the laccase. *Applied and Environmental Microbiology*.

[41] Sharma K. K., Shrivastava B., Sastry V. R. B., Sehgal N., Kuhad R. C. (2013). Middle-redox potential laccase from Ganoderma sp.: its application in improvement of feed for monogastric animals. *Scientific Reports*.

[42] Rajendran K., Annuar M. S. M., Karim M. A. A. (2011). Medium optimization for fungal laccase production. *Asia Pacific Journal of Molecular Biology and Biotechnology*.

[43] D'Souza T. M., Merritt C. S., Reddy C. A. (1999). Lignin-modifying enzymes of the white rot basidiomycete *Ganoderma lucidum*. *Applied and Environmental Microbiology*.

[44] Park J.-W., Kang H.-W., Ha B.-S., Kim S.-I., Kim S., Ro H.-S. (2015). Strain-dependent response to Cu^2+^ in the expression of laccase in *Pycnoporus coccineus*. *Archives of Microbiology*.

[45] Baldrian P. (2003). Interactions of heavy metals with white-rot fungi. *Enzyme and Microbial Technology*.

[46] Palmieri G., Giardina P., Bianco C., Fontanella B., Sannia G. (2000). Copper induction of laccase isoenzymes in the ligninolytic fungus *Pleurotus ostreatus*. *Applied and Environmental Microbiology*.

[47] Couto S. R., Gundín M., Lorenzo M., Sanromán M. Á. (2002). Screening of supports and inducers for laccase production by *Trametes versicolor* in semi-solid-state conditions. *Process Biochemistry*.

[48] You L.-F., Liu Z.-M., Lin J.-F., Guo L.-Q., Huang X.-L., Yang H.-X. (2014). Molecular cloning of a laccase gene from *Ganoderma lucidum* and heterologous expression in *Pichia pastoris*. *Journal of Basic Microbiology*.

[49] Murugesan K., Nam I.-H., Kim Y.-M., Chang Y.-S. (2007). Decolorization of reactive dyes by a thermostable laccase produced by *Ganoderma lucidum* in solid state culture. *Enzyme and Microbial Technology*.

[50] Leitner C., Hess J., Galhaup C. (2002). Purification and characterization of a laccase from the white-rot fungus Trametes multicolor. *Applied Biochemistry and Biotechnology—Part A Enzyme Engineering and Biotechnology*.

[51] Morozova O. V., Shumakovich G. P., Gorbacheva M. A., Shleev S. V., Yaropolov A. I. (2007). ‘Blue’ laccases. *Biochemistry*.

